# Treatment of Acute Rheumatism

**Published:** 1880-06-20

**Authors:** J. Alexander Larue

**Affiliations:** W. Virginia


### TREATMENT OF ACUTE RHEUMATISM.

BY J. ALEXANDER LARUE, M.D., OF W. VIRGINIA.

Those who advocate the salicylic acid treatment *alone*, should remember that in ignoring the alkaline treatment they deny the pathology of the disease. That acute articular rheumatism is due to an acid poison in the blood is a fact generally admitted; and admitting the fact, the alkaline treatment seems to be the most scientific and reasonable of any yet presented to the profession.

Although it may be said that the salicylic acid treatment does shorten the attack, yet it has been the experience of some of our most successful and scientific practitioners that where the salicylic acid alone was used, the liability to endocarditis and pericarditis was greatly increased. On the other hand, the alkaline treatment does not, perhaps, shorten the duration of the attack, but it does diminish the liability to those heart troubles so frequently the sequela of that painful disease.

It seems to me, therefore, that a combination of the salicylic acid with some alkali would best meet the indications. I give my prescription, hoping thereby to add something, at least, for suffering humanity.

℞ Acid salicylic......... ........................................ grs. clx.

Bicarb. Sodæ.................................................. grs. cccxx.

Aqua................................................................ ℥ iv. M.

Dose, teaspoonful every three hours. Should heart-trouble arise, I find great benefit from :

℞ Tinc. verat.-virid., (Norwoods)..............................

Tinc. digitalis..................................................aa ʒ ij. M

Dose, six to eight drops every six hours. I have found this combination of verat. virid. and digitalis of especial benefit in valvular lesions.

At the beginning of an attack of acute articular rheumatism, the system seems to be "locked up," and I always advise a free cathartic, and in my opinion mercury is the best, in a majority of cases, because there seems to be a decided inactive or torpid liver.

That great benefit results from the use of blisters there is no doubt in my mind, and in extreme cases I have always used them, and always was satisfied with their good effects.

However robust and strong a patient may have been previous to an attack of acute rheumatism, the system is always left in a debilitated condition.

Tonics are necessary, and I do not know that any is superior to mur. tinct. of iron, or if preferred in another form :

℞ Quiniæ and ferri cit ........................................ grs. clx.

Ext. nucis vomic...... .........................................fl ʒ iij.

Simp. syr. q. s. to make........................................ ℥ iv. M

Ft. sol. One teaspoonful three times a day; or :

℞ Ferri sulph. ex...............................................
Quiniæ sulph.................................................aa grs. xxx.
Ex. nucis vomic. (solid.)................. ...................... grs. x.

Ft. pills No. 30. Dose, one pill three times daily.—*South Clinic.*

